# Study Profile: The Japan “Society and New Tobacco” Internet Survey (JASTIS): A Longitudinal Internet Cohort Study of Heat-Not-Burn Tobacco Products, Electronic Cigarettes, and Conventional Tobacco Products in Japan

**DOI:** 10.2188/jea.JE20180116

**Published:** 2019-11-05

**Authors:** Takahiro Tabuchi, Tomohiro Shinozaki, Naoki Kunugita, Masakazu Nakamura, Ichiro Tsuji

**Affiliations:** 1 Cancer Control Center, Osaka International Cancer Institute, Osaka, Japan; 2 Department of Biostatistics, School of Public Health, the University of Tokyo, Tokyo, Japan; 3 Department of Environmental Health, National Institute of Public Health, Saitama, Japan; 4 Health Promotion Research Center, Institute of Community Medicine, Japan Association for Development of Community Medicine, Tokyo, Japan; 5 School of Health Sciences, University of Occupational and Environmental Health, Fukuoka, Japan; 6 Division of Epidemiology, Department of Health Informatics & Public Health, Tohoku University School of Public Health, Graduate School of Medicine, Miyagi, Japan

**Keywords:** The Japan “Society and New Tobacco” Internet Survey (JASTIS), longitudinal internet cohort study, heat-not-burn tobacco products, electronic cigarettes, conventional tobacco products, Japan

## Abstract

**Background:**

Japan became the first country where heat-not-burn tobacco products were sold. Therefore, there was no information for actual status on the actual use status or the harms of heat-not-burn tobacco products. The objectives of the study profile are to generate data that can be freely available to external researchers, and to create collaborative research projects in the future.

**Methods:**

The Japan “Society and New Tobacco” Internet Survey (JASTIS) is a longitudinal internet cohort study which investigates perception, attitude, and use of heat-not-burn tobacco, electronic cigarettes (e-cigarettes), and conventional tobacco products in Japan. The survey also includes demographic, health-related, and socioeconomic factors. Participants were randomly selected and invited from internet panelists. The baseline survey was closed when the target number of respondents who had answered the questionnaire was met.

**Results:**

The study includes three cohorts (1–3) from the 2015 baseline survey and a cohort (4) from the 2017 baseline survey: cohorts 1 and 4 were recruited based on sex and age: men and women aged 15–69 years (*n* = 8,240 for cohort 1 and *n* = 5,897 for cohort 4); cohorts 2 and 3 were created using status-based recruiting: e-cigarette and/or heat-not-burn tobacco ever users (*n* = 2,188; cohort 2) and combustible cigarette smokers without e-cigarette/heat-not-burn tobacco experience (*n* = 724; cohort 3). The completion rates were 8.5% to 9.9%. All subjects were followed and assessed annually. Response rates for the follow-up survey were 65.5% in 2016, 55.3% in 2017, and 50.9% in 2018. Because Internet-based responders are not a representative sample of the general population of Japan, we conducted adjustment to account for “being an internet survey respondent” and reported tobacco product use in Japan. A recent JASTIS study reported that prevalence of IQOS current-use among Japanese adults had rapidly increased from 0.3% in 2015 to 3.6% in 2017.

**Conclusion:**

The JASTIS study provides the first estimates for heat-not-burn tobacco use in the world and e-cigarette use in Japan. For information on collaboration, please contact the corresponding author.

## BACKGROUND AND PURPOSE

Electronic cigarettes (e-cigarettes) are battery-operated devices that contain an inhalation-activated mechanism that heats a cartridge, producing aerosol. Since their introduction to the market in 2004, e-cigarettes have become popular, especially among adolescents and young adults in North American and European countries.^1^^–^^3^ In Japan, e-cigarettes with nicotine liquid have been prohibited by the Pharmaceutical Affairs Act since 2010, but non-nicotine e-cigarettes were available to the public, even to minors, because there was no regulation for non-nicotine e-cigarettes in Japan. Under these circumstances, e-cigarette use was not popular in Japan. In December 2013, an international tobacco company, Japan Tobacco, began online sales, in Japan, of a new heat-not-burn tobacco product, “Ploom”, that vaporizes tobacco leaf. Furthermore, in 2014 Philip Morris International introduced a novel heat-not-burn tobacco product, “IQOS”, which heats specific tobacco leaf sticks, in Japan and Italy. Therefore, Japan became the only country where two new brands of heat-not-burn tobacco products were sold in 2014. As these products are new to the world, it is not surprising that there is no information on the actual use status or the harms of heat-not-burn tobacco products.

E-cigarettes have been marketed to consumers as a less harmful alternative to conventional tobacco smoking.^4^ Some researchers consider e-cigarettes to be safer for both users and bystanders than conventional tobacco smoking, with the potential to reduce the burden of smoking-related diseases and death.^3^^,^^5^^,^^6^ However, the actual health effects on users, the efficacy of e-cigarettes for cessation, and the overall impact of e-cigarettes on public health (tobacco control policies) are still under debate.^7^^–^^10^ A number of research questions concerning e-cigarettes remain unanswered about heat-not-burn tobacco products.

Therefore, the Japan “Society and New Tobacco” Internet Survey (JASTIS), launched in 2015, was specifically designed to estimate the prevalence of use of novel heat-not-burn tobacco and e-cigarettes in Japan and to investigate any association or causal link between novel product use and behavioral changes, such as combustible cigarette cessation and relapse. The aims of the study are listed in Table 1. An important aim of the study is to monitor and observe tobacco product use status among users. Specifically, there has been concern about the impact of new product use on existing tobacco control measures: eg, e-cigarette or cigarette-like product use in smoke-free areas will make enforcement of smoke-free policies more complicated and undermine social norms that discourage tobacco smoking.^11^^,^^12^ Moreover, there are several additional specific concerns about heat-not-burn tobacco and e-cigarettes. For example, we examined whether the educational gradient of e-cigarette/heat-not-burn tobacco use was different from that of conventional cigarette smoking.^13^ The missions of the JASTIS study are to develop evidence with research publications and disseminate information on heat-not-burn tobacco-related issues from Japan, which has, unfortunately, become an experimental field for heat-not-burn tobacco products. The objectives of the study profile are to communicate the information of this research project, to inform that data can be freely used by external researchers, and to create many collaborative studies in the future.

**Table 1.** Aims, study population and study examples in the JASTIS study

Aims		Study population (Cohort) for aim	Findings		

			Specific theme	Analyzed data	Publications
1	To estimate prevalence of tobacco product use among general population^a^	Cohort 1	Prevalence of e-cigarette and heat-not-burn tobacco products use in Japan	Cohort 1	Tabuchi et al 2016 & Tabuchi et al 2018

2	To monitor tobacco product use status among users	Cohort 2, 3 and reconstructed users from cohort 1, 2 and 3	Actual e-cigarette/heat-not-burn tobacco use in smoke-free places	Cohort 2 (current and former regular users)	Kiyohara et al 2018

3	To observe behavior changes such as smoking cessation, relapse and gateway effect	Cohort 1, 2 and 3 with both cross-sectional and longitudinal assessments	Association between e-cigarette/ heat-not-burn tobacco use and smoking cessation	Retrospective analysis of cohort 1 (case-control study design)	Hirano et al 2017

4	Specific concerns and others	All	Educational gradient of e-cigarette/ heat-not-burn tobacco use	Cohort 1	Miyazaki et al 2018

			Association between tobacco products use and chronic diseases	Cohort 1	Kioi et al 2018

			Smoke-free status of home and car	Cohort 1	Shojima et al 2019

			Association between exposure to tobacco company promotion and preferred smoke-free policy	Cohort 1	Prepared

^a^Estimated by inverse probability weights calculated from JASTIS cohorts and a representative sample of Japan’s general population (details are explained in the “Strengths and Limitations” section).

## METHODS

### Participants

The study used a prospective cohort design. Participants were recruited from a large survey panel managed by a major, nationwide internet research agency, Rakuten Insight (former Rakuten Research), which maintains a pool of 2.3 million panelists covering all social categories, such as education, housing tenure, and marital status, defined by the census in Japan.^14^ The survey panel consisted of people recruited initially through services managed by the Rakuten agency group. At the time of registration, participants were required to provide information, such as sex, age, occupation, and area of residence, and to agree that they would participate in different research surveys with web-based written consent. Minors provided their consent with approval from their parents or guardians.

The study consisted of three cohorts from the 2015 baseline survey (see Table 2) and a cohort from the 2017 baseline survey (see Table 3): cohort 1 (sex and age-based recruiting) enrolled men and women aged 15–69 years (*n* = 8,240); cohort 2 (use status-based recruiting) enrolled e-cigarette and/or heat-not-burn tobacco ever users (*n* = 2,188); cohort 3 (use status-based recruiting) enrolled combustible cigarette smokers without e-cigarette/heat-not-burn tobacco experience (*n* = 724); and cohort 4 (sex and age-based recruiting) enrolled men and women aged 15–69 years (*n* = 5,897). Cohort 1 and 4 participants were randomly selected from the total panel members, whereas cohort 2 and 3 participants were randomly selected from 32,179 adult panelists (aged 20–69 years) who had previously reported product use in previous surveys conducted by the Rakuten Research in 2013 and 2014. We aimed to collect data from (1) 9,000 panelists (500 people aged 15–19 years and 800 people aged 20–29, 30–39, 40–49, 50–59, and 60–69 years for both sexes), (2) 2,400 panelists who had ever used e-cigarettes (800 current, 800 former regular, and 800 former non-regular users), (3) 800 panelists who were current smokers of combustible cigarettes without e-cigarette/heat-not-burn tobacco experience, and (4) 6,000 panelists (150 people aged 15–19 years, about 640 people aged 20–29, 30–39 and 40–49 years, and 470 people aged 50–59 and 60–69 years for both sexes). The survey was closed when the target number of respondents who had answered all the questionnaire items was met. Because there were few teenage panelists, target numbers for 15–19 years were set as 500 people (cohort 1) and 150 people (cohort 4). Because follow-up rates were lower in young adults, target numbers for young adults were set higher (cohort 4).

**Table 2.** Number of participants in the JASTIS study (Cohort 1, 2 and 3)

Characteristics at baseline	Wave 1	Wave 2	Wave 3	Wave 4

	January 31 to February 17, 2015	January 29 to February 15, 2016	January 27 to February 27, 2017	January 26 to March 20, 2018

	Number of participants	Respondents of follow-up survey (%)	Respondents of follow-up survey (%)	Respondents of follow-up survey (%)
Cohort 1: designation only for sex and age	8,240	5,366 (65.1)	4,217 (51.2)	3,873 (47.0)
men, 15–19 years old	443	95 (21.4)	51 (11.5)	42 (9.5)
men, 20–29 years old	720	429 (59.6)	305 (42.4)	274 (38.1)
men, 30–39 years old	728	507 (69.6)	410 (56.3)	375 (51.5)
men, 40–49 years old	740	571 (77.2)	467 (63.1)	443 (59.9)
men, 50–59 years old	722	550 (76.2)	458 (63.4)	439 (60.8)
men, 60–69 years old	731	610 (83.4)	511 (69.9)	489 (66.9)
women, 15–19 years old	438	128 (29.2)	86 (19.6)	78 (17.8)
women, 20–29 years old	742	384 (51.8)	274 (36.9)	240 (32.3)
women, 30–39 years old	737	454 (61.6)	369 (50.1)	309 (41.9)
women, 40–49 years old	747	522 (69.9)	407 (54.5)	363 (48.6)
women, 50–59 years old	739	542 (73.3)	416 (56.3)	382 (51.7)
women, 60–69 years old	753	574 (76.2)	463 (61.5)	439 (58.3)

Cohort 2: e-cigarette/heat-not-burn tobacco ever users	2,188	1,387 (63.4)	1,480 (67.6)	1,375 (62.8)
current user	727	405 (55.7)	505 (69.5)	462 (63.5)
former regular user	727	464 (63.8)	506 (69.6)	464 (63.8)
former non-regular user	734	518 (70.6)	469 (63.9)	449 (61.2)

Cohort 3: smokers without e-cigarette/ Heat-not-burn tobacco use experience	724	547 (75.6)	470 (64.9)	433 (59.8)

↓ Sample reconstruction				

Sum of cohort 1, 2 and 3	11,152	7,300 (65.5)	6,167 (55.3)	5,681 (50.9)

Combustible cigarette smoking category				
current smoker	3,279	2,276 (69.4)	2,078 (63.4)	1,945 (59.3)
former smoker	2,301	1,559 (67.8)	1,356 (58.9)	1,262 (54.8)
never smoker	5,572	3,465 (62.2)	2,733 (49.0)	2,474 (44.4)

E-cigarette/Heat-not-burn tobacco category				
current user	779	431 (55.3)	521 (66.9)	475 (61.0)
former user	1,849	1,208 (65.3)	1,152 (62.3)	1,071 (57.9)
never user	8,524	5,661 (66.4)	4,494 (52.7)	4,135 (48.5)

Response rates in parentheses. Numbers of participants after exclusion of cases with data discrepancies.

**Table 3.** Number of participants in the JASTIS study (Cohort 4)

Characteristics at baseline	Wave 1	Wave 2

	February 24 to March 13, 2017	January 26 to March 20, 2018

	Number of participants	Respondents of follow-up survey (%)
Cohort 4 (additional new baseline from 2017): designation only for sex and age	5,897	4,641 (78.7)
men, 15–19 years old	144	60 (41.7)
men, 20–29 years old	582	398 (68.4)
men, 30–39 years old	657	528 (80.4)
men, 40–49 years old	628	543 (86.5)
men, 50–59 years old	462	411 (89.0)
men, 60–69 years old	467	428 (91.7)
women, 15–19 years old	148	54 (36.5)
women, 20–29 years old	628	445 (70.9)
women, 30–39 years old	628	473 (75.3)
women, 40–49 years old	629	514 (81.7)
women, 50–59 years old	462	383 (82.9)
women, 60–69 years old	462	404 (87.5)

Response rates in parentheses. Numbers of participants after exclusion of cases with data discrepancies.

### Variables

Table 4 provides a detailed overview of the measures included in each wave. For example, panelists were asked about their use in the previous 30 days of each new product (e-cigarettes, Ploom, IQOS, and glo) in each survey (glo was included from 2017, because it entered the market in December 2016). The term “Ploom TECH” was used instead of Ploom from 2017, following the product change. The following question was used: “Have you used the following products in the previous 30 days?”.^15^^,^^16^ Questions had been included to monitor the trends of product use, but this depended on the study budget (few variables in 2016). Furthermore, questionnaires had been also modified by the emergence of new research questions. The Japanese questionnaires are available upon request to the corresponding author.

**Table 4.** Collected information in waves

Category	Content	Wave 1: 2015	Wave 2: 2016	Wave 3: 2017	Wave 4: 2018
			
		Baseline	Follow-up	Follow-up New baseline	Follow-up
Tobacco-related	Tobacco product use status	○	○	○	○
	Combustible cigarette	○	○	○	○
	e-cigarette with nicotine	○	○	○	○
	e-cigarette without nicotine	○	○	○	○
	IQOS	○	○	○	○
	Ploom/Ploom Tech	○	○	○	○
	glo			○	○
	Other tobacco products	○	○	○	○
	Tobacco product use in previous 1 year		○	○	○
	Tobacco product use in previous 30 days	○	○	○	○
	Cigarette/puff per day	○	○	○	○
	Awareness of e-cigarette/HnB tobacco products	○			
	Awareness and perception of tobacco control policies including FCTC	○		○	○
	Preference regarding e-cigarette use (would you like to use e-cigarette in future?)	○			
	Smoke-free rules at workplace, home and car	○		○	○
	Attitudes towards tobacco smoking	○		○	○
	Perceptions against e-cigarette/HnB tobacco harm	○		○	○
	e-cigarette/HnB tobacco use in smoke-free places	○		○	○
	Trial of smoking cessation	○	○	○	○
	Reasons to use e-cigarette/HnB tobacco	○	○	○	○
	Smoking cessation stage	○	○	○	○
	Symptoms from own e-cigarette/HnB tobacco use	○	○	○	○
	Experience of exposure to secondhand smoking			○	○
	Experience of exposure to secondhand e-cigarette/HnB tobacco aerosol and perceived symptoms from the exposure	○		○	○
	Preferred smoke-free policy			○	○
	Awareness of tobacco-related events in Japan			○	○
	Exposure to tobacco related information such as posters, TVCM and books			○	○
	Exposure to and perception of tobacco company promotion			○	○
	Perceptions to tobacco packaging in Japan			○	○
Demographic	Sex	○	○	○	○
	Age	○	○	○	○
	Area of residence	○		○	○
	Housing tenure	○		○	○
	Marital status	○		○	○
	Number of household members	○		○	○
Socioeconomic	Education	○		○	○
	Income	○		○	○
	Health insurance	○		○	○
	Occupation	○		○	○
Health related	Alcohol drinking status	○	○	○	○
	Drug use status	○	○	○	○
	Self-rated health	○		○	○
	Chronic diseases and hospital visit	○		○	○
	Happiness scale			○	○
Others	Exposure to mass media such as television and newspaper			○	○

Abbreviations: FCTC, Framework Convention on Tobacco Control; HnB, heat-not-burn; TVCM, television commercial.

### Statistical analysis

Respondents of an internet study are not representative of the general population, so we conducted statistical adjustment to account for bias. Harmonization of the data with a major national and representative cross-sectional study would allow us to pool data, providing the potential capacity to adjust for “being a respondent in an internet survey”.^17^^,^^18^ Since this method cannot completely adjust for the difference in respondents between an internet survey and a nationwide representative survey, the problem of generalizability remains. However, this method could approximate our estimate to a nationally representative estimate, using inverse probability weighting to account for baseline characteristics, such as socio-demographic, health-related, and tobacco-related factors.^17^^,^^18^ Details have been given in previous reports.^15^^,^^18^

The response rate in the follow-up survey was also problematic, given that non-responders differ in a number of ways from the respondents in the survey, and there was evidence to suggest that attrition was higher among the younger and smoking populations. Therefore, to account for potential non-random non-response, an additional adjustment for “non-response in the follow-up survey” was conducted, giving inverse probability weighting to the remaining participants in each survey by modeling the probability of not dropping out.^19^

Furthermore, we excluded respondents showing discrepancies and/or artificial/unnatural responses in the analyses. For example, choosing the same number all the time in a set of questions was used to detect a discrepancy. Details for discrepancy are also shown in previous reports.^15^^,^^18^

After these adjustments and exclusions, characteristics of the baseline study subjects in cohort 1 in 2015 are shown in Table 5. Of the total baseline subjects, 50% were male, 59% were never-smokers, and 6% were ever-users of heat-not-burn tobacco/e-cigarette in 2015.^18^

**Table 5.** Characteristics of baseline study subjects (Cohort 1 in 2015)

Characteristics at baseline 2015	*n*^a^	%
**Total**	8,240	100.0
**Sex**		
Men	4,084	49.6
Women	4,156	50.4
**Age groups, years**		
15–19	881	10.7
20–29	1,462	17.7
30–39	1,465	17.8
40–49	1,487	18.1
50–59	1,461	17.7
60–69	1,484	18.0
**Smoking status**		
Never-smoker	4,839	58.7
Former smoker	1,582	19.2
Current smoker with intention to quit	281	3.4
Current smoker with no intention to quit	1,538	18.7
**Workplace indoor smoking ban status**		
No ban (including smoking room/corner)	2,699	32.8
Complete ban	3,256	39.5
Not working/did not know	2,285	27.7
**Heat-not-burn-tobacco/e-cigarette use status**		
Never used with no preference for e-cigarette use	7,369	89.4
Never used, but would like to try e-cigarette in future	353	4.3
Ever-user	518	6.3
**Equivalent household income**		
1st quartile (Lowest)	2,079	25.2
2nd quartile	1,646	20.0
3rd quartile	1,531	18.6
4th quartile (Highest)	1,256	15.2
Did not know/did not want to answer	1,729	21.0
**Housing tenure**		
Does not own housing	2,237	27.2
Owns housing	6,003	72.9
**Education**		
Junior high school/high school	4,905	59.5
University/technical school/college or higher	3,335	40.5
**Marital status**		
Married	4,970	60.3
Never married	2,802	34.0
Divorced/widowed	468	5.7
**Alcohol consumption**		
Never-drinker	3,360	40.8
Former drinker	473	5.8
Current drinker	4,406	53.5
**Self-rated health**		
Good (excellent/very good/good)	7,356	89.3
Poor (fair/poor)	884	10.7

^a^Adjusted for “being a respondent in an internet survey” using weights to make the cohorts representative of the national general population in Japan.

## RESULTS

The participation rate is defined as the number of respondents who have provided an eligible response divided by the total number of initial personal invitations requesting participation.^20^ However, as the internet research agency does not know whether panelists recognize the invitation (e-mail) or not, only the final number of participants for the survey is available. Furthermore, because the internet survey finishes when the target sample size is reached, the participation rate was calculated to be low; eg, if the target sample size is 1,000 and the invitation is sent to 100,000 panelists, the participation rate must be low, 1.0% (1,000/100,000). The participation rate (completion rate) was calculated to be 8.5% (9,055/106,202) for cohort 1 and 9.9% (3,201/32,179) for cohorts 2 and 3 at the end of the 2015 survey. Due to the nature of the internet survey, respondents were skewed to those who had responded earlier.

The four completed annual waves are shown in Table 2 (numbers of participants after exclusion of cases with data discrepancies). A follow-up survey was conducted every year from the last Friday of January to February or March. All subjects were assessed annually. Response rates for the follow-up survey are presented in parentheses in Table 2. Of the 11,152 participants (sum of cohorts 1, 2, and 3), 3,852 (34.5%) withdrew or were lost to further follow-up, so 7,300 respondents remained in 2016 (total response rate, 65.5%). Respective total response rates in 2017 and 2018 were 55.3% and 50.9%. Distribution of respondents in follow-up surveys was adjusted close to baseline distribution using weights. The characteristics of study subjects, after this adjustment, are shown in a supplementary table in a previous paper.^15^ Because the number of respondents in the sample decreased due to non-response to follow-up surveys, an additional baseline survey was conducted in 2017 (cohort 4: *n* = 5,897; Table 3; numbers of participants after exclusion of cases with data discrepancies). Annual follow-up surveys and additional baseline surveys will be conducted according to the budget of our research group.

### Findings and publications

Several articles have been published on the Japan “Society and New Tobacco” Internet Survey (Table 1).^13^^,^^15^^,^^18^^,^^21^^–^^23^ Using the 2015 baseline data with a cross-sectional design (cohort 1), Tabuchi et al^18^ reported the prevalence of awareness and use of e-cigarettes and heat-not-burn tobacco products in Japan. This was the first study to report the actual use of heat-not-burn tobacco products in the world and e-cigarettes in Japan. In 2015, 48.0% (95% confidence interval [CI], 46.9–49.1%) of respondents were aware of e-cigarettes and/or heat-not-burn tobacco products, 6.6% (95% CI, 6.1–7.1%) had ever used at least one e-cigarette and/or heat-not-burn tobacco product, 72.3% (95% CI, 68.6–76.1%) of ever-users used non-nicotine e-cigarettes, and 33.4% (95% CI, 29.5–37.4%) of them used nicotine e-cigarettes. Ploom and iQOS were used by 7.8% (95% CI, 5.5–10.0%) and 8.4% (95% CI, 6.1–10.7%), respectively, of ever-users, with a relatively higher share among the younger population.

Since e-cigarettes and heat-not-burn tobacco products have been marketed to consumers as an aid for smoking cessation, regardless of the truth of such claims, we need to investigate the effect of e-cigarettes and heat-not-burn tobacco products on combustible cigarette cessation. Using the 2015 baseline data with a case-control study design (798 eligible persons who smoked 5 years ago in cohort 1), Hirano et al^22^ examined the association between e-cigarette use and smoking cessation, compared with nicotine replacement therapy, smoking cessation therapy, and unassisted cessation. E-cigarette use was negatively associated with smoking cessation (odds ratio [OR] 0.63; 95% CI, 0.41–0.96) after adjusting for sex, age, health-related factors, and other quitting methods. Conversely, smoking cessation therapy (ie, varenicline) was significantly associated with smoking cessation (OR 1.89; 95% CI, 1.02–3.49) in the same model. In this analysis, e-cigarette use appears to have low efficacy on smoking cessation, but further prospective investigation using a longitudinal analysis is necessary.

In addition to the prevalence of use and relationship with smoking cessation, the influence of novel tobacco product use on smoke-free policies has been a concern.^10^ The original aim of smoke-free policies was to protect non-smokers from secondhand smoke. However, smoke-free policies have also been instrumental in de-normalizing smoking behavior, as well as lowering smoking prevalence.^24^ Since e-cigarettes have also been marketed to consumers as a means of evading smoke-free policies,^25^^,^^26^ the use of e-cigarettes in places where conventional tobacco smoking is prohibited could potentially re-normalize tobacco smoking, sustain the dual use of e-cigarettes and tobacco, maintain nicotine addiction, and complicate enforcement of smoke-free policies.^11^^,^^12^^,^^27^ Kiyohara et al^21^ investigated the actual use of e-cigarettes in public places where conventional tobacco smoking is not permitted. Among adult Japanese e-cigarette ever-users (current and former regular users in cohort 2), approximately 26–29% had ever used and 16–19% had frequently used e-cigarettes in restaurants and workplaces where combustible tobacco smoking is not allowed.

Recently, Tabuchi et al^15^ reported the results of 1- and 2-year follow-up surveys, which were conducted in 2016 (wave 2) and 2017 (wave 3), and updated the prevalence of e-cigarettes and heat-not-burn tobacco use in Japan. In 2015, 1.3% (95% CI, 1.1–1.6%) of respondents (both sexes) were current e-cigarette users (use in the previous 30 days), while 0.3% (95% CI, 0.2–0.4%) were current IQOS users and, similarly, 0.3% (95% CI, 0.2–0.4%) were Ploom users. By 1 year later, in 2016, these levels had not changed greatly: 1.4% (95% CI, 1.2–1.7%) for e-cigarettes, 0.6% (95% CI, 0.5–0.8%) for IQOS and 0.3% (95% CI, 0.1–0.4%) for Ploom. In 2017, the e-cigarette current-user rate had slightly increased to 1.9% (95% CI, 1.6–2.2%), while the IQOS current-user rate had increased considerably to 3.6% (95% CI, 3.2–4.0%). The Ploom current-user rate also increased, but only to 1.2% (95% CI, 0.9–1.4%), and the glo current-user rate was 0.8% (95% CI, 0.6–1.0%) in 2017. A popular television program triggered IQOS diffusion in Japan.^15^

Importantly, the prevalence of heat-not-burn tobacco use dramatically increased in 2017.^15^ Monitoring the tobacco epidemic is the foundation of successful tobacco control.^28^ As no representative national study has measured heat-not-burn tobacco use in Japan, continued monitoring of novel tobacco product use and the publication of articles are important tasks for the JASTIS research project.

### Strengths and limitations

The study provides four areas of innovation. First, it provides the first assessment of the actual use of heat-not-burn tobacco products in the world. This is because Japan is the first country where IQOS has been rolled-out nationally, and Japan’s worldwide share of IQOS was more than 90% in October 2016.^29^ After the baseline survey, Japan Tobacco launched a new product “Ploom TECH” in March 2016 and British American Tobacco also began to sell a new heat-not-burn tobacco product, “glo”, in December 2016 in Japan. Second, therefore, the study also provides a comprehensive assessment of three heat-not-burn tobacco products, IQOS, Ploom TECH and glo, in addition to other products, such as combustible cigarettes, e-cigarettes, cigars, and snus, allowing comparison across a wide spectrum of product use. Third, the study is also the first to longitudinally assess behavior changes for multiple tobacco product use, including heat-not-burn tobacco products. To date, nothing is known about the impact of heat-not-burn tobacco use on combustible cigarette smoking cessation. Moreover, probabilities of switching to other products and using multiple products concurrently are also unknown about heat-not-burn tobacco products. This information will improve our understanding of the total influence of heat-not-burn tobacco products and suggest options for regulation of the products for policy makers. Fourth, the study will provide a basis for epidemiological research into various specific concerns around tobacco problems, such as the association between chronic disease diagnosis and heat-not-burn tobacco use^23^ (Table 1). A new question about heat-not-burn tobacco use will be added to national survey questionnaires in the future, but at the moment the study is one of the few important data sources.

A major focus of the study was on comprehensive (prospective) data capture for tobacco-related issues in Japan. Given the low prevalence of some tobacco product use (other than major tobacco products) in Japan, it is unlikely that the study will be able to provide a validated estimate, especially for non-popular products. This is due to the smaller size of the sample. Finally, the information collected was self-reported and is, thus, subject to potential biases. Although a no-bias value was not available, in order to address this limitation, we excluded respondents with discrepancies from the analyses. Despite these limitations, the study will improve understanding of the current situation regarding all tobacco and related products, including heat-not-burn tobacco, in Japan, which can direct health policy. The results can inform development of public health prevention and early intervention campaigns to allow people to make informed choices about heat-not-burn tobacco use.

### Information on collaboration

The dataset is freely available for external researchers on the following collaborative study framework. Data access is governed by the investigators. Research proposals must be consistent with ethical approval, confidentiality, and data management. A list of research themes is maintained by the research group. The study protocol for collaborative research requires consent from the respective research groups affiliated with the research. Further information can be obtained through Dr Tabuchi (corresponding author) at the Cancer Control Center, Osaka International Cancer Institute.
