# Erratum to ‘Study Profile: The Japan “Society and New Tobacco” Internet Survey (JASTIS): A Longitudinal Internet Cohort Study of Heat-Not-Burn Tobacco Products, Electronic Cigarettes, and Conventional Tobacco Products in Japan’

**DOI:** 10.2188/jea.JE20190317

**Published:** 2020-01-05

**Authors:** Takahiro Tabuchi, Tomohiro Shinozaki, Naoki Kunugita, Masakazu Nakamura, Ichiro Tsuji

**Affiliations:** 1 Cancer Control Center, Osaka International Cancer Institute, Osaka, Japan; 2 Department of Biostatistics, School of Public Health, the University of Tokyo, Tokyo, Japan; 3 Department of Environmental Health, National Institute of Public Health, Saitama, Japan; 4 School of Health Sciences, University of Occupational and Environmental Health, Fukuoka, Japan; 5 Health Promotion Research Center, Institute of Community Medicine, Japan Association for Development of Community Medicine, Tokyo, Japan; 6 Division of Epidemiology, Department of Health Informatics & Public Health, Tohoku University School of Public Health, Graduate School of Medicine, Miyagi, Japan

In the above article, there were errors in authors’ affiliations. The correct affiliations are as shown in bold below.

^1^Cancer Control Center, Osaka International Cancer Institute, Osaka, Japan
^2^Department of Biostatistics, School of Public Health, the University of Tokyo, Tokyo, Japan
^3^Department of Environmental Health, National Institute of Public Health, Saitama, Japan
**^4^School of Health Sciences, University of Occupational and Environmental Health, Fukuoka, Japan**
**^5^Health Promotion Research Center, Institute of Community Medicine, Japan Association for Development of Community Medicine, Tokyo, Japan**
^6^Division of Epidemiology, Department of Health Informatics & Public Health, Tohoku University School of Public Health, Graduate School of Medicine, Miyagi, Japan

